# PnpM, a LysR-Type Transcriptional Regulator Activates the Hydroquinone Pathway in *para*-Nitrophenol Degradation in *Pseudomonas* sp. Strain WBC-3

**DOI:** 10.3389/fmicb.2017.01714

**Published:** 2017-09-14

**Authors:** Jin-Pei Wang, Wen-Mao Zhang, Hong-Jun Chao, Ning-Yi Zhou

**Affiliations:** ^1^Wuhan Institute of Virology, Chinese Academy of Sciences Wuhan, China; ^2^University of Chinese Academy of Sciences Beijing, China; ^3^State Key Laboratory of Microbial Metabolism and School of Life Sciences & Biotechnology, Shanghai Jiao Tong University Shanghai, China

**Keywords:** catabolism, hydroquinone pathway, LysR-type transcriptional regulator, *para*-nitrophenol, *Pseudomonas* sp. strain WBC-3, PnpM, PnpR

## Abstract

A LysR-type transcriptional regulator (LTTR), PnpR, has previously been shown to activate the transcription of operons *pnpA, pnpB*, and *pnpCDEFG* for *para*-nitrophenol (PNP) degradation in *Pseudomonas* sp. strain WBC-3. Further preliminary evidence suggested the possible presence of an LTTR additional binding site in the promoter region of *pnpCDEFG*. In this study, an additional LTTR PnpM, which shows 44% homology to PnpR, was determined to activate the expression of *pnpCDEFG*. Interestingly, a *pnpM*-deleted WBC-3 strain was unable to grow on PNP but accumulating hydroquinone (HQ), which is the catabolic product from PNP degradation by PnpAB and the substrate for PnpCD. Through electrophoretic mobility shift assays (EMSAs) and promoter activity detection, only PnpR was involved in the activation of *pnpA* and *pnpB*, but both PnpR and PnpM were involved in the activation of *pnpCDEFG*. DNase I footprinting analysis suggested that PnpR and PnpM shared the same DNA-binding regions of 27 bp in the *pnpCDEFG* promoter. In the presence of PNP, the protection region increased to 39 bp by PnpR and to 38 bp by PnpM. Our data suggested that both PnpR and PnpM were involved in activating *pnpCDEFG* expression, in which PNP rather than the substrate hydroquinone for PnpCD is the inducer. Thus, during the PNP catabolism in *Pseudomonas* sp. strain WBC-3, *pnpA* and *pnpB* operons for the initial two reactions were controlled by PnpR, while the third operon (*pnpCDEFG*) for HQ degradation was activated by PnpM and PnpR. This study builds upon our previous findings and shows that two LTTRs PnpR and PnpM are involved in the transcriptional activation of these three catabolic operons. Specifically, our identification that an LTTR, PnpM, regulates *pnpCDEFG* expression provides new insights in an intriguing regulation system of PNP catabolism that is controlled by two regulators.

## Introduction

It is well-known that versatile bacterial strains swiftly adapt and respond to polluted environments. One such adaptation is based on modulation of gene expression, particularly those encoding bacterial catabolism of pollutants. Various transcriptional regulation systems play an important role in adaptive responses (Cases and de Lorenzo, 2001; Shingler, 2003). Regulatory proteins are the key elements that control the transcription of catabolic operons to ensure the successful establishment of a catabolic pathway (Diaz and Prieto, 2000). The LysR-type transcriptional regulators (LTTRs) are the most abundant in prokaryotes, controlling diverse bacterial functions, including stress response, motility, antibiotic resistance, quorum sensing, aromatic compound degradation, and amino-acid biosynthesis (Schell, 1993; Maddocks and Oyston, 2008). Tetramers are the active form of LTTRs, and their interaction with the promoter regions generally occurs at two dissimilar sites: regulatory binding site (RBS) and activation binding site (ABS). The RBS contains an LTTR consensus binding motif (T-N_11_-A) and is usually centered near position −65 relative to the transcriptional start site of the activated promoter. While ABS, for which no conserved sequence motif has been identified thus far, is located near position −35. Although LTTRs are capable of binding DNA in the absence of inducer molecules, transcriptional activation of downstream genes requires inducers (Schell, 1993; Diaz and Prieto, 2000; Tropel and van der Meer, 2004; Maddocks and Oyston, 2008).

**Figure 1:**
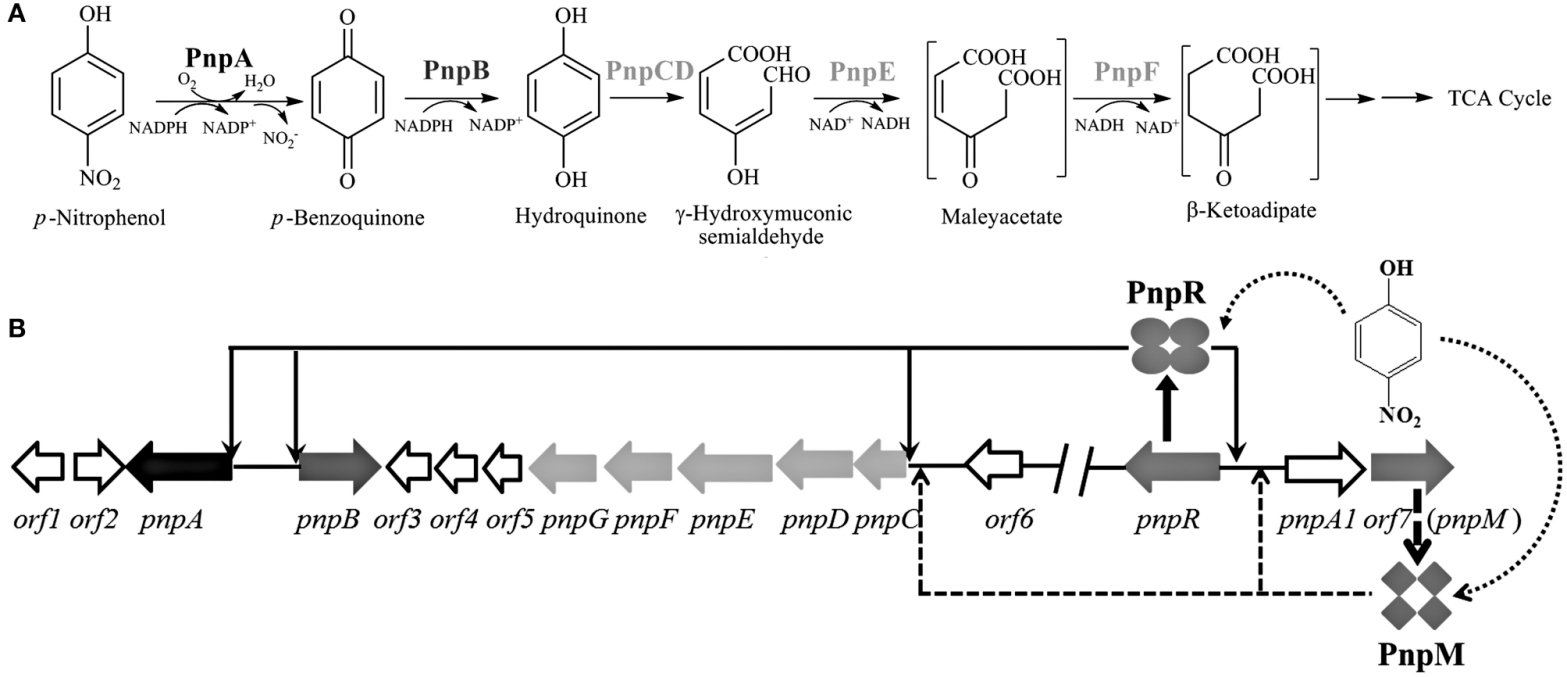
**(A)** Proposed pathway for PNP catabolism in *Pseudomonas* sp. strain WBC-3 (modified after Zhang et al., 2009) *pnpA* and *pnpB* are PNP 4-monooxygenase and *p*-benzoquinone reductase respectively; *pnpCDEF* encode α- and β-subunits of hydroquinone dioxygenase, γ-hydroxymuconic semialdehyde dehydrogenase and maleylacetate reductase, respectively. **(B)** Schematic of the regulatory circuit of the PNP catabolic cluster in *Pseudomonas* sp. strain WBC-3 (figure not drawn to scale, modified after Zhang et al., 2015). Four ellipses stand for PnpR tetramer, and four diamonds stand for PnpM tetramer. The curved and dotted arrow stands for binding of *para*-nitrophenol (PNP) with PnpR or PnpM. The down arrows stand for PnpR's activating four operons of *pnpA, pnpB, pnpCDEFG*, and *pnpR*. The up arrows stand for PnpM's activating two operons of *pnpCDEFG* and *pnpA1M*.PnpR regulates transcription initiation in four regions *pnpA, pnpB, pnpCDEFG*, and *pnpR*. PnpM regulates transcription initiation in two regions *pnpCDEFG* and *pnpA1M*.

As a typical representative of mononitrophenols, *para*-nitrophenol (PNP) is toxic to humans and is widely utilized in the chemical syntheses of pharmaceuticals, dyes and pesticides (Karim and Gupta, 2002). So far, a number of bacterial strains capable of utilizing PNP have been isolated and their diverse catabolic pathways have been elucidated (Spain and Gibson, 1991; Jain et al., 1994; Roldan et al., 1998; Arora et al., 2014). *Pseudomonas* sp. strain WBC-3 utilizes PNP as its sole source of carbon, nitrogen, and energy. Strain WBC-3 metabolizes PNP *via* the hydroquinone (HQ) pathway and the genes involved in the PNP catabolism are located on three different operons: *pnpA* (encoding PNP 4-monoxygenase catalyzing monooxygenation of PNP to *p*-benzoquinone), *pnpB* [encoding *p*-benzoquinone (BQ) reductase converting *p*-benzoquinone to HQ], and *pnpCDEFG* (encoding the enzymes catalyzing the conversion of HQ to β-ketoadipate) (Zhang et al., 2009), as shown in Figure 1A. PnpR, encoded by a gene at least 16 kb away from the PNP catabolic operons, is a LTTR and found to be involved in the regulation of all three operons (Zhang et al., 2015). In other PNP utilizers, regulatory proteins were also proposed to be involved in PNP catabolism: (1) LTTR PnpR regulated HQ degradation based on the gene knock-out in *Pseudomonas putida* DLL-E4 (Shen et al., 2010); and (2) AraC-type regulator NphR regulated PNP oxidation based on the gene mutation and transcriptional activity analysis in Gram-positive *Rhodococcus* sp. strain PN1 (Takeo et al., 2008).

Our previous work has shown that PnpR regulates all four operons of *pnpR, pnpA, pnpB* and *pnpCDEFG* in strain WBC-3 (Zhang et al., 2015). The promoter activities of *pnpR, pnpA*, and *pnpB* were completely lost after mutation in their corresponding RBSs. Surprisingly, no changes in promoter activity occurred between the mutant and wild type RBS of the promoter of the *pnpCDEFG* operon. This data suggested that the *pnpCDEFG* promoter possibly has evolved from a different origin, evidenced by a longer distance (20 nucleotides) between −35 box and RBS of *pnpCDEFG* promoter than those (9–10 nucleotides) of the other three promoters. Therefore, one of the reasons for this phenomenon was speculated to be an additional regulator involved in the activation of *pnpCDEFG* operon (Zhang et al., 2015). In this study, we confirmed that indeed an additional LTTR PnpM, encoded by a gene close to *pnpR*, was involved in the regulation of *pnpCDEFG* operon for HQ degradation in PNP degradation by strain WBC-3.

## Materials and methods

### Bacterial strains, chemicals, culture media, and DNA manipulations

The bacterial strains and their relevant genotypes are described in Table 1. *Pseudomonas* sp. strain WBC-3 was cultivated at 30°C in lysogeny broth (LB) or minimal medium (MM) with 0.5 mM of PNP as the sole source of carbon and nitrogen. Bacto agar was added to a final concentration of 15 g L^−1^ for the solid media. When necessary, antibiotics and other additions were used at the following concentrations: ampicillin (100 mg L^−1^), chloramphenicol (34 mg L^−1^), gentamicin (20 mg L^−1^), kanamycin (100 mg L^−1^), tetracycline hydrochloride (10 mg L^−1^) gentamicin (10 mg L^−1^), and ONPG (*o*-Nitrophenyl β-D-galactopyranoside, 4 mg L^−1^). Reagents were from Fluka Chemical Co. (Buchs, Switzerland) or Sigma Chemical Co. (St. Louis, MO).

**Table 1:** Bacterial strains and plasmids in this study.

**Bacterial strain**	**Genotype/phenotype**	**References or source**
*Pseudomonas* sp. strain WBC-3	PNP and methyl parathion utilizer, wild type	Chen et al., 2002
WBC3-Δ*pnpM*	WBC-3 mutant with *pnpM* gene disrupted	This study
WBC3-Δ*pnpM*C	WBC3-Δ*pnpM* with *orf 6* replaced by *pnpM*	This study
*Pseudomonas putida* PaW340	Mxy^−^ Mtol^−^ PNP^−^ Str^r^ Trp^−^	Williams and Murray, 1974; Liu et al., 2005b
***E. coli*** **STRAINS**		
DH5α	λ^−^Φ80d*lacZ*ΔM15 Δ(*lacZYA-argF*) U169 *recA*1 *endA*1 *hsdR*17 (${\text{r}}_{\text{k}}^{-}$ ${\text{m}}_{\text{k}}^{+}$) *supE*44 *thi*-1 *gyrA relA*1	Woodcock et al., 1989
BL21	F^−^*ompT hsdS*(${\text{r}}_{\text{B}}^{-}$${\text{m}}_{\text{B}}^{+}$) *gal dcm*(DE3)	Jeong et al., 2009
WM3064	*thrB1* 004 *pro thi rpsL hsdS lacZ*ΔM15 RP4-1360Δ (*araBAD*)*567* Δ*dapA*1341::[*erm pir*(wt)]	Saltikov and Newman, 2003
**PLASMIDS**		
pMD18-T	Amp^r^, *lacZ*; cloning vector	Takara
pEX18Tc	Tc^r^ *sacB*^+^, gene replacement vector	Hoang et al., 1998
pEX18Gm	Gm^r^; *oriT*^+^*sacB*^+^, gene replacement vector with MCS from pUC18	Hoang et al., 1998
pTn*Mod*-OKm	Km^r^, source of neomycin phosphotransferase II gene (*nptII*)	Dennis and Zylstra, 1998
pEX18Tc-*kanpnpM*	pEX18Tc with a 1.0 kb PCR fragment carrying the flanking regions of *pnpM* disrupted by *nptII*	This study
pKO*orf6*-Gm*pnpR*	pEX18Tc with a 1.0 kb PCR fragment carrying the flanking regions of *orf6* disrupted by *aacC1* and *pnpR*	Zhang et al., 2015
pKO*orf6*-*gmpnpM*	pEX18Tc with a 1.0 kb PCR fragment carrying the flanking regions of *orf6* disrupted by *aacC1* and *pnpM*	This study
pEX18Tc-*cmgfp*	Tc^r^, pEX18Tc with *cm* gene and *gfp* gene	Hu et al., 2014
pCM*gfp*-*lacZ*	pCM*gfp* containing *lacZ*	Zhang et al., 2015
pCM*gfp*-spC*lacZ*	spC-*lacZ* translational fusion in pCM*gfp* carrying the sequence between positions −94 and +25	Zhang et al., 2015
pCM*gfp*-lpC*lacZ*	lpC-*lacZ* translational fusion in pCM*gfp* carrying the sequence between positions −255 and +25	Zhang et al., 2015
pCM*gfp*-spCm*lacZ*	pCM*gfp*-spC*lacZ* with GTT of RBS to AAA	Zhang et al., 2015
pET30a(+)	Expression vector, Km^r^, C/N-terminal His•Tag/thrombin/T7•Tag, T7 *lac* promoter, T7 transcription start, f1 origin, *lacI*	Novagen
pET30a-*pnpM*	pET30a containing a 1 kb fragment *pnpM* with C-terminal His•Tag	This study
pBBR1mcs-2	Km^r^, broad host range, *lacPOZ*′	Kovach et al., 1995
pVLT33	Km^r^, RSF 1010-*lacl*q/P*tac* hybrid broad-host-range expression vector, MCS of pUCl8	de Lorenzo et al., 1993
pBBR1-*tacpnpR*	pBBR1mcs-2 containing a 1 kb fragment *pnpR* (his-tag sequence at its 3′ end) and promoter *tac*	Zhang et al., 2015
pBBR1-*tacexpnpM*	pBBR1mcs-2 containing a 1 kb fragment *pnpM* (his-tag sequence at its 3′ end) and promoter *tac*	This study
pBBR1-*tacpnpMpnpR*	pBBR1mcs-2 containing a 1 kb fragment *pnpM*, a 1 kb fragment *pnpR* and promoter *tac*	This study

Plasmids used are summarized in Table 1, and the primers used are described in Table S1. All DNA manipulations were performed according to standard procedures (Sambrook and Maniatis, 1989). Restriction enzymes, DNA polymerases and T4 DNA ligase were used according to the manufacturers' specifications.

### Gene knockout and complementation

The *pnpM-*knockout plasmid pEX18Tc-*kanpnpM* was constructed by fusion of the upstream and downstream fragments of *pnpM* that was amplified from genomic DNA of strain WBC-3 and the kanamycin resistance gene amplified from plasmid pTn*Mod*-Okm (Dennis and Zylstra, 1998) to pEX18Tc (Hoang et al., 1998) with In-Fusion HD Cloning Kit (Clontech, Beijing, China). The construct pEX18Tc-*kanpnpM* was then transformed into *E. coli* WM3064 before it was conjugated with strain WBC-3 as previously described (Dehio and Meyer, 1997; Saltikov and Newman, 2003). The WBC-3Δ*pnpM* double-crossover recombinant was screened on LB plates with 15% (wt/vol) sucrose and 50 mg of L^−1^ kanamycin.

The entire *pnpM* together with its promoter region P*pnpA1* and gentamycin resistant gene *aacC1* was cloned into pKO*orf6* (Zhang et al., 2015) using an In-Fusion kit, yielding construct pKO*orf6*-*gmpnpM* for *pnpM* complementation. The *pnpM* complemented strain WBC-3Δ*pnpM*C was obtained by the process of *pnpM* knockout, with the exception that 50 mg L^−1^ kanamycin was replaced by 10 mg L^−1^ gentamicin.

The growth of WT strain WBC-3 and its derivatives on 0.3 mM PNP or HQ, and their capability of utilizing the substrates was determined by monitoring their growth as well as the substrate consumption. The growth curves were fitted with modified Gompertz equation (Zwietering et al., 1990) with OriginPro 8 software.

### Biotransformation

Biotransformation was performed as previously described (Chen et al., 2014) with slight modifications. Strain WBC-3Δ*pnpM* was grown in 100 ml MM containing 5% LB to an optical density at 600 nm (OD_600_) of 0.6 and then induced with 0.3 mM PNP for 6 h. The cultures were collected for high-performance liquid chromatography (HPLC) analyses, by a described method (Min et al., 2014). The specific activity is defined as the weight of cells required to convert 1 μM of substrate per minute at 30°C.

### RNA preparation and real-time quantitative PCR (RT-qPCR)

Total RNA was purified with an RNAprep Pure Kit for Bacteria (Tiangen Biotech, Beijing, China), and reverse transcribed into cDNA with a PrimeScript RT Reagent kit (TaKaRa, Dalian, China). RT-qPCR was performed using the CFX96TM Real-Time PCR Detection System (Bio-Rad, Hercules, CA) following the iQ SYBR Green Supermix (Bio-Rad) manufacturer's recommendation. The expression levels of all of the genes were normalized to 16S rRNA gene expression as an internal standard and quantified according to a reported method (Livak and Schmittgen, 2001). All experiments were performed in triplicate.

### 5′ rapid amplification of cDNA ends (5′-RACE)

The transcriptional start site (TSS) of the *pnpA1M* operon was determined using a SMARTer RACE cDNA amplification kit (Clontech, Mountain View, CA). First-strand cDNA was synthesized with a PrimeScript RT Reagent kit (TaKaRa, Dalian, China), and the cDNA was then performed with terminal transferase and dCTP for homopolymeric tailing. The tailed cDNA was then amplified using the abridged anchor primer (AAP) and pnpA1M-GSP2. Finally, this product was used as a template for nested PCR with AAP and primer pnpA1M-GSP3 and then cloned into pMD18-T (TaKaRa) for sequencing.

### Expression and purification of PnpM

*pnpM* was amplified from genomic DNA of strain WBC-3 and was cloned into pET-30a. The resulting plasmid pET30a-*pnpM* was transformed into *E. coli* BL21 (DE3) pLysS and purified as described (Liu and Zhou, 2012). PnpM purified from *E. coli* was demonstrated to be nonfunctional in subsequent experiments, and *Pseudomonas putida* strain PaW340, which is a mutant derivative of *m*-toluate/*m*-xylene utilizer *Pseudomonas putida* mt-2 and usually used as an expression host (Williams and Murray, 1974). It worthies to mention that *Pseudomonas putida* mt-2 and *Pseudomonas putida* PaW340 are not PNP utilizers (Liu et al., 2005b), and then the strain PaW340 was chosen as the alternative expression host.

Subsequently, the DNA fragment *tac* promoter (P*tac*) amplified from plasmid pVLT33 (de Lorenzo et al., 1993) and the *pnpM* gene were cloned into pBBR1mcs-2 to construct the plasmid pBBR1-*tacexpnpM*. In addition, DNA fragments of P*tac, pnpM* and *pnpR* were also cloned into pBBR1mcs-2 to construct the plasmid pBBR1-*tacpnpMpnpR*. The construct pBBR1mcs2-*tacexpnpM* was introduced into *Pseudomonas putida* strain PaW340. C-terminal His-tagged PnpM (PnpM-His_6_) was expressed and purified as previously described (Zhang et al., 2015). The concentration of purified PnpM-His_6_ was calculated using the Protein Assay Kit (Beyotime Co., Shanghai, China). The protein was stored in glycerol at −20°C.

### Gel filtration chromatography

The sample containing PnpM-His_6_ was loaded onto a column (1.6 × 60 cm) of Hiload Superdex 200 pg (FPLC system, GE Healthcare, Little Chalfont, UK) that was equilibrated with 10 mM of Tris-HCl buffer (pH 7.5, with 0.1 M NaCl and 5% glycerol) for 2 h at a flow rate of 0.5 ml min^−1^. The target protein was then eluted with the same buffer. The native molecular mass of PnpM was estimated from a calibration curve plotted by using the standard proteins (Zhang et al., 2009).

### Electrophoretic mobility shift assays (EMSAs)

Electrophoretic Mobility Shift Assay (EMSA) was performed as previously described (Georgi et al., 2008) with minor modifications. DNA fragments containing respective promoters of *pnpA, pnpB, pnpC*, and *pnpA*1 were obtained by PCR with genomic DNA of strain WBC-3 as templates. Different amounts (0–2.0 μM) of purified PnpM-His_6_ were used in each reaction, respectively. Two DNA fragments (0.03 μM each), with approximately 400 bp respectively of *gfp* gene amplified from pEX18Tc-cmgfp (Hu et al., 2014), were used as the control DNA. After 30 min of incubation at room temperature, the samples were then loaded onto a 6% native polyacrylamide gel at 4°C and electrophoresed in Tris-borate buffer for 1.5 h at 130 V. Subsequently, the gels were stained with SYBR green I (BioTeKe Co., Beijing, China). DNA and DNA–protein complexes were visualized using an UltraBright LED Transilluminator (Maestrogen, Taiwan, China).

### DNase I footprinting assay

Footprinting assays were performed as previously described (Zianni et al., 2006). A 270 bp DNA fragment of promoter *pnpC* was cloned into pUC18H (TOLO Biotech, Shanghai) in preparation of the probe. The correct construct was used as the template for further preparation of fluorescent 6-carboxyfluorescein (FAM)-labeled probes. The samples for DNase I footprinting were conducted as for the EMSAs. For each assay, the sample of binding reaction was treated as previously described (Zhang et al., 2015). The digested DNA was examined by a DNA Analyzer (Applied Biosystems, Waltham, MA, USA) and the GeneScan-LIZ500 standard size (Applied Biosystems) was used. The data analysis was the same as previously described (Wang et al., 2012).

### Promoter activity analysis

β-Galactosidase assays were employed to analyze the expression of promoter-*lacZ* fusions in the surrogate host *Pseudomonas putida* PaW340, which is incapable of degrading PNP (Liu et al., 2005b). The activity was determined by SDS- and chloroform-permeabilized cells as described (Griffith and Wolf, 2002; Zhang et al., 2015).

### Statistical analysis

Statistical analysis was performed using Origin 8.0 software. Paired-samples tests were used to calculate probability values (*p*) of the transcription of *pnpA, pnpC*, and *pnpM*. Paired-samples tests and one-way analysis of variance (ANOVA) was used to calculate the probability values (P) for β-galactosidase activity analyses of *pnpA, pnpC*, and promoters. *P*-values of < 0.05 and < 0.01 were considered to be significant and highly significant, respectively.

## Results

### *pnpM* is essential in HQ degradation

As hypothesized in the introduction, an additional regulator was probably involved in *pnpCDEFG* operon activation. The previously designated *orf7* (Zhang et al., 2015) (*orf* WBC3-000012) from contig 001 (accession number KM019215) of strain WBC-3, right at the downstream of *pnpA1* (*orf* WBC3-000011), was found to encode a LysR-type regulatory protein (accession number: AIV98012) (Figure 1B). The *orf7* was re-designated *pnpM* and its product was 44% identical to PnpR that had previously been demonstrated to be involved in the four operons for PNP catabolism.

**Figure 2:**
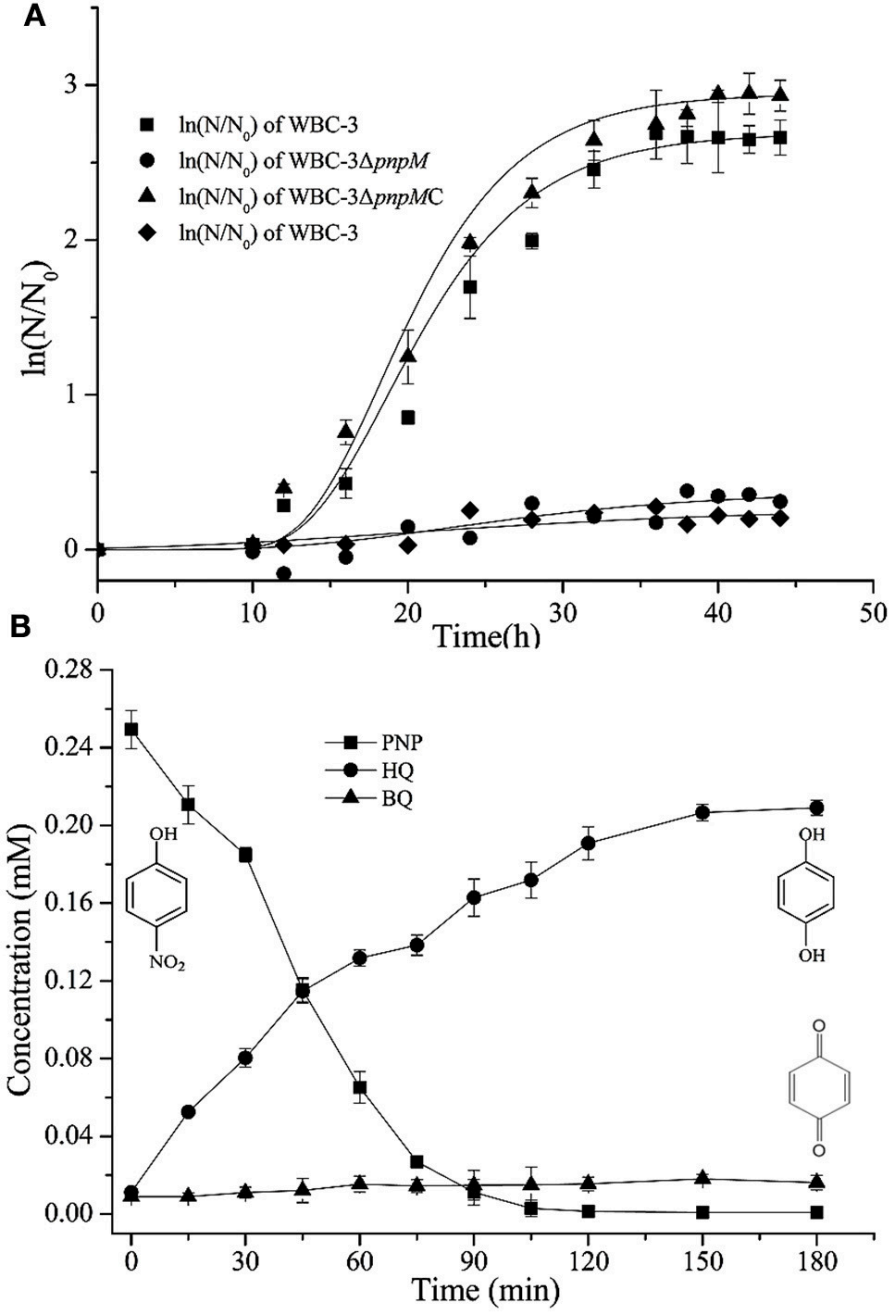
**(A)** Growth curve of strains WBC-3, WBC-3Δ*pnpM*, and WBC-3Δ*pnpM*C. Three strains were grown at 30°C for 44 h in MM containing 0.3 mM PNP, and strain WBC-3 was also grown in MM containing 0.3 mM HQ. The curves were fitted by the modified Gompertz model with Origin software. The values are averages of three independent experiments. Error bars indicate standard deviations. N, number of cells; N_0_, initial number of cells. Square (■) stands for strain WBC-3. Circle (•) stands for strain WBC-3Δ*pnpM*. Triangle (▴) stands for strain WBC-3Δ*pnpM*C that the gene *pnpM* is complemented into the genome of strain WBC3-Δ*pnpM*. Diamond (♦) stands for strain WBC-3 grown on MM containing 0.3 mM HQ. **(B)** Time course of PNP degradation by strain WBC-3Δ*pnpM* in whole-cell biotransformation. Samples were withdrawn at the time points indicated and treated immediately as described in the text. The disappearance of PNP and the appearance of the products (BQ and HQ) were quantified by HPLC. The experiments were performed in triplicate, the results shown are average values of three independent experiments, and error bars indicate standard deviations.

To investigate the possible regulatory role of PnpM in triggering catabolic operons for PNP degradation of strain WBC-3, *pmpM* was knocked out. The obtained mutant strain WBC3-Δ*pnpM* was no longer able to grow on PNP (Figure 2A) but could still transform PNP (yellow-green) to a brown substance without further degradation (test III in Figure 2B). This accumulated product was determined to be HQ by HPLC analysis. The insertion of plasmids in strain WBC-3 is technically challenging (Liu et al., 2005a), therefore, strain WBC3-Δ*pnpM* was complemented by inserting *pnpM* into the *orf6* locus of mutant strain WBC3-Δ*pnpM* through a double cross-over. The complemented strain WBC3-Δ*pnpM*C, where *pnpM* replaced *orf6*, is able to grow on PNP (Figure 2A). Meanwhile, the wild-type strain WBC-3 was incapable of growing on HQ as the sole source of carbon (Figure 2A). However, PNP-induced cells of strain WBC-3 could degrade HQ with a specific activity of 0.78 ± 0.23 U mg^−1^. In a biotransformation experiment by strain WBC3-Δ*pnpM*, PNP consumption (249 μM) was approximately equivalent to the total accumulation of HQ (210 μM) and BQ (18 μM) (Figure 2B), indicating a nearly stoichiometric formation of HQ and BQ from PNP. These results suggested that PnpM was likely a positive regulator for the *pnpCDEFG* operon encoding enzymes for HQ degradation in PNP catabolism.

### Transcriptional analysis of PNP catabolic operons

**Figure 3:**
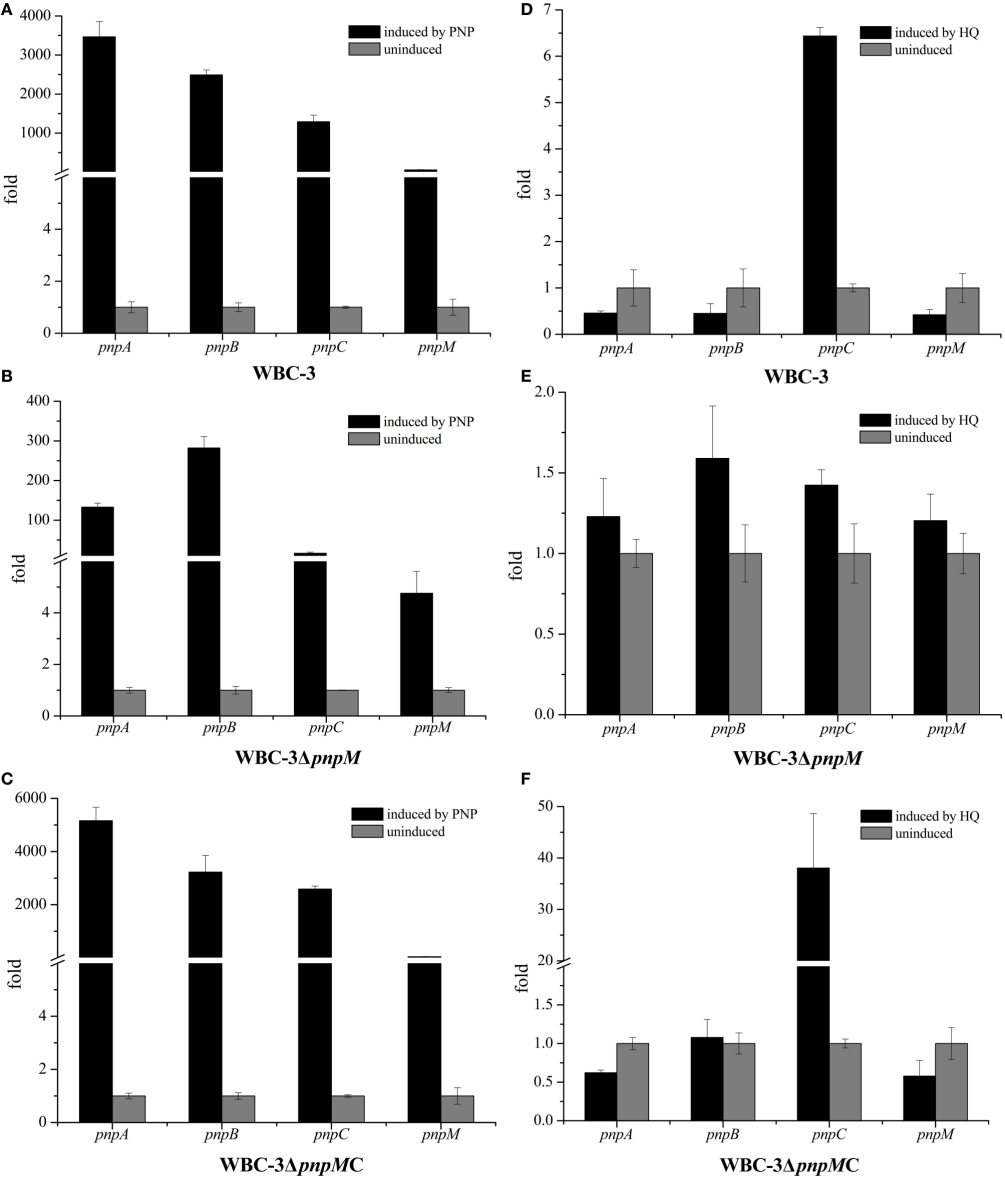
Transcriptional analysis of *pnpA, pnpB, pnpCDEFG*, and *pnpA1M* operons in wild type strain WBC-3 **(A,D)**, *pnpM* knockout strain WBC-3Δ*pnpM* **(B,E)** and complemented *pnpM*-knockout strain WBC-3Δ*pnpM*C **(C,F)**. The levels of gene expression in each sample were calculated as the fold expression ratio after normalization to 16S rRNA gene transcriptional levels. The values are averages of three independent qRT-PCR experiments. Error bars indicate standard deviations. There was a significant difference in the transcription of *pnpA, pnpB, pnpC*, and *pnpM* between strains WBC-3 and WBC-3Δ*pnpM* (*p* < 0.01, Paired-samples test), respectively. Black columns stand for the fold of gene expression level induced by PNP, and gray columns stand for the fold of gene expression level without PNP.

To investigate the possible regulatory role on the transcriptional expression of the entire *pnp* gene cluster, the impact of PnpM was further analyzed by determining the transcriptional levels of all three PNP catabolic operons, as well as *pnpA1M* operon in both the WT strain and the mutant strain WBC-3Δ*pnpM*. When PNP is present, the transcriptional levels of four tested genes (each representing one of the four operons, *pnpA, pnpB, pnpCDEFG*, and *pnpA1M* as shown in Figure 1B) in WT strain WBC-3 were enhanced dramatically compared to the absence of PNP, with the transcriptional levels of *pnpA, pnpB, pnpC*, and *pnpM* being 3,465-, 2,490-, 1,292-, and 56-fold higher (Figure 3A), respectively, through qRT-PCR assay. The increased level of the three catabolic operons was at the same order of magnitude in WT strain WBC-3 from above figures, but the increased level of *pnpA* (130-fold) and *pnpB* (282-fold) in strain WBC-3Δ*pnpM* was about >10-fold that of *pnpC* (15-fold) (Figure 3B). In the *pnpM-*complemented strain WBC-3Δ*pnpM*C, the transcriptional levels of the three genes were similar to those of corresponding genes in the WT strain WBC-3 under the same conditions (Figure 3C). These results indicated that *pnpM* deletion had a greater impact on the transcription of *pnpC* than *pnpA* and *pnpB*.

Similarly, the transcriptional levels of these genes were also analyzed under HQ induction conditions. In the presence of HQ, the transcriptional level of *pnpC* was increased in the WT strain and the *pnpM-*complemented strain (Figures 3D,F), but was significantly reduced in the presence of PNP (Figures 3A,C); while no change of the transcription level of *pnpA, pnpB*, and *pnpM* was observed between the induced and non-induced conditions (Figures 3D,F). In strain WBC3-Δ*pnpM*, the transcriptional levels of *pnpA, pnpB*, and *pnpC* were almost unaltered with or without HQ (Figure 3E). These results indicated that PnpM served as an activator for *pnpC* transcriptional expression under PNP induction.

### PnpM is a tetramer

In order to obtain a functional regulator for *in vitro* assays, PnpM-His_6_ was overexpressed as a C-terminal His-tagged fusion protein from the pBBR1-*tacexpnpM* (a broad-host-range vector-based construct) in *Pseudomonas putida* PaW340, which is a mutant derivative of *m*-toluate/*m*-xylene utilizer *Pseudomonas putida* mt-2 and usually used as an expression host (Williams and Murray, 1974). It worthies to mention that *Pseudomonas putida* mt-2 and *Pseudomonas putida* PaW340 are not PNP utilizers (Liu et al., 2005b). The predicted size of the monomer of PnpM, 35 kDa, was confirmed by SDS-PAGE (Figure S1). By analytical gel filtration, the homologously produced PnpM-His_6_ was eluted as a single peak corresponding to 150 kDa (Figure S2) in the chromatography buffer with or without PNP. This was consistent with PnpM as a tetramer.

### PnpM binds with the promoter region of *pnpC*

The transcription start site (TSS) of *pnpC* was previously identified by 5′-RACE to be located to a T nucleotide, 23 nt upstream of the *pnpC* translation start codon (Zhang et al., 2015), as shown in **Figure 5C**. In this study, *pnpA1* and *pnpM* were confirmed to be located in the same operon by RT-PCR. The TSS of *pnpA1M* operon was shown to be an A nucleotide, 54 nt upstream of the translation start codon of *pnpA1*. The putative −35 and −10 regions of the *pnpC* and *pnpM* promoters were proposed as shown in **Figures 5C,D**.

**Figure 4:**
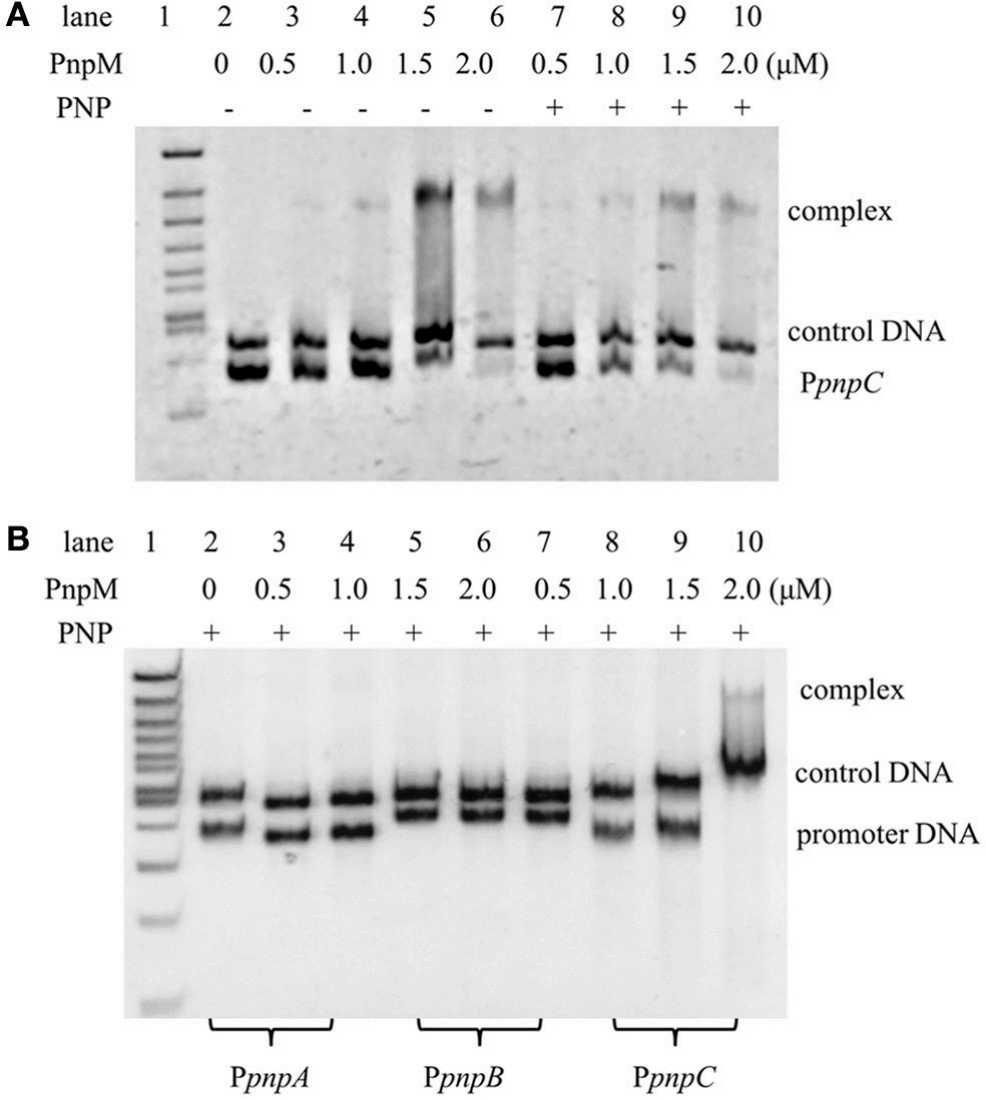
Electrophoretic mobility shift assays of PnpM binding with *pnp* promoters. **(A)** Electrophoretic mobility shift assays of PnpM binding with *pnpC* promoter (P*pnpC*). PnpM binding with P*pnpC* with PNP or without PNP. +, Stands for with PNP, −, stands for without PNP. The first lane was 100 bp ladder Marker, lanes 2–6 contain 0.03μM DNA, with 0μM, 0.5μM, 1.0μM, 1.5μM, and 2.0μM PnpM, respectively; lanes 7–10 contain 0.03μM DNA and 0.3 mM PNP, with 0.5μM, 1.0μM, 1.5μM, and 2.0μM PnpM, respectively. An approximately 400 bp DNA fragment *gfp* was used as a control DNA (0.03μM). Free probe is a fragment P*pnpC* of about 270 bp. **(B)** PnpM binding with *pnp* promoters in the present of PNP. The first lane was 100 bp ladder Marker, lanes 2–4 contain 0.03μM 283 bp DNA fragment of P*pnpA* and 0.3 mM PNP, with 0μM, 1.0μM, and 2.0μM PnpM, respectively; lanes 5–7 contain 0.03μM 334 bp DNA fragment of P*pnpB* and 0.3 mM PNP, with 0μM, 1.0μM, and 2.0μM PnpM, respectively; lanes 8–10 contain 0.03μM 270 bp DNA fragment of P*pnpC* and 0.3 mM PNP, with 0μM, 1.0μM, and 2.0μM PnpM, respectively. An approximately 400 bp DNA fragment *gfp*-1 was used as a control DNA (0.03μM).

As previously reported, PnpR was capable of specifically binding the promoter of operons *pnpA, pnpB, pnpCDEFG*, and *pnpR*. It is worthy to note that, during a pre-experiment, PnpM in the range of 0–2μM was initially employed to bind with P*pnpC* (Figure 4A), where the DNA was found to be completely bound with 2.0 μM PnpM and this capacity was not changed with or without PNP. Here, EMSA was employed to assess the possible regulation of *pnpA, pnpB, pnpC* expression by PnpM. After, purified PnpM-His_6_ was incubated with the promoter regions of *pnpA* (P*pnpA*), *pnpB* (P*pnpB*), and *pnpC* (P*pnpC*) in the presence of PNP, PnpM was shown to specifically bind directly to the promoter region of *pnpCDEFG*, but not to the other promoters (Figure 4B). In addition, PnpM could bind to the promoter region of *pnpA1M* (Figure S3).

### Footprinting analysis of PnpM and PnpR binding site to *pnpC* promoter

**Figure 5:**
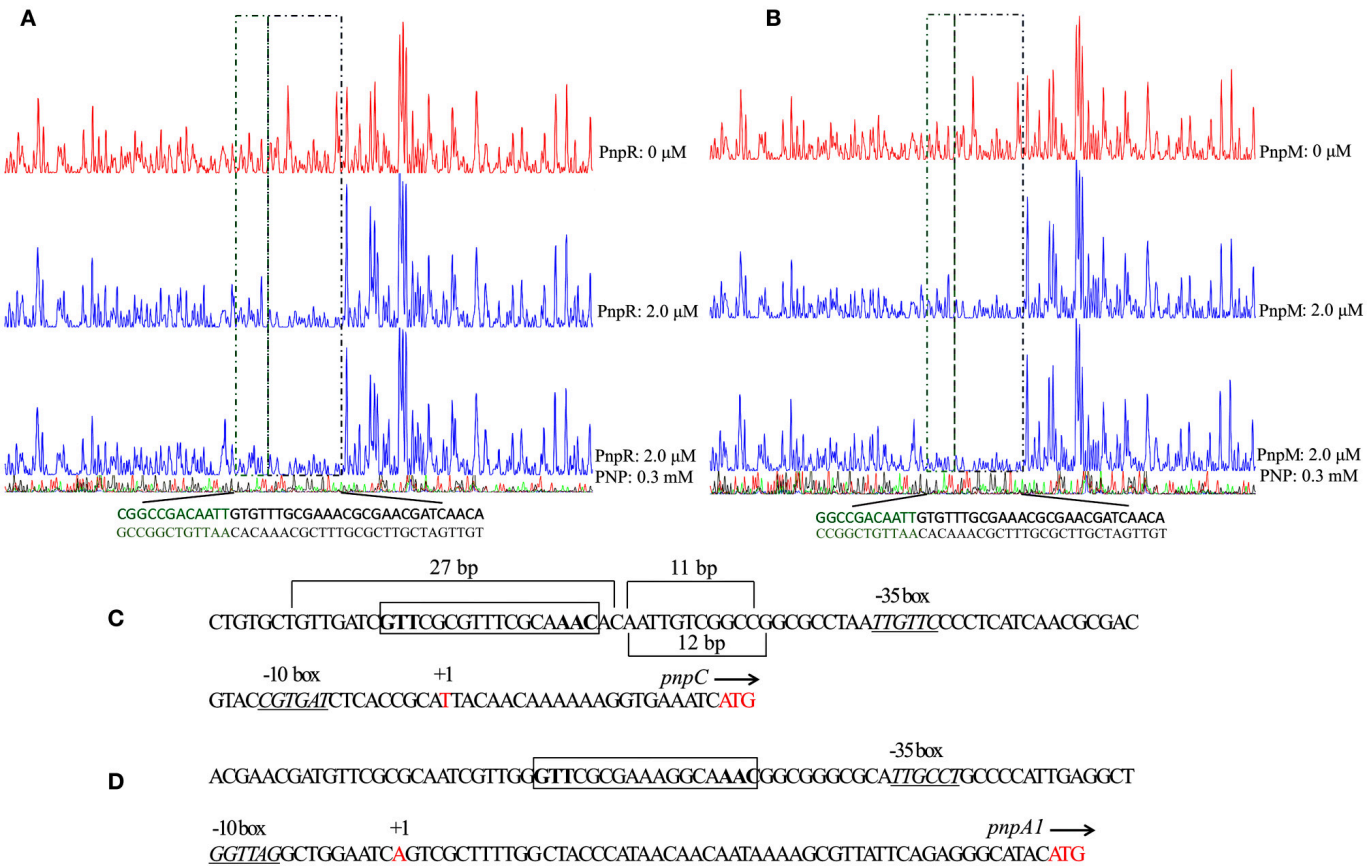
The DNase I footprinting analysis of PnpR **(A)** or PnpM **(B)** binding to P*pnpC* with PNP or without PNP. An amount of 0.03 μM probe P*pnpC* covering the entire intergenic region of *pnpC* was incubated with 2.0 μM PnpM in the EMSA buffer with or without PNP (0.3 mM). The intergenic fragment was labeled with 6-carboxyfluorescein (FAM) dye, incubated with 2.0 μM PnpM (blue line) or without PnpM (red line). The region protected without PNP by PnpM from DNase I cleavage are indicated with a black dotted box. The extended protected region with PNP are indicated with a green dotted box. PnpR with P*pnpC* and PNP was treated in the same way of PnpM. The organizations of upstream regions of *pnpCDEFG* **(C)** and *pnpA1M* **(D)** operons. The putative -10 boxes and -35 boxes are underlined and in italics. Start codon ATG and TSSs are in red. TSSs are denoted by arrow and +1, and the direction of arrow stands for the direction of gene transcription. The putative regulatory binding sites (RBSs) GTT-N_11_-AAC are boxed. The promoter regions of *pnpCDEFG* protected from DNase I digestion by PnpM without and with PNP. The protected region is 27 bp by PnpM or PnpR without PNP. When PNP was present, a region of 11 bp of the protection region was increased by PnpM, while a region of 12 bp by PnpR.

DNase I footprinting was employed to determine the specific DNA motif within the *pnpC* promoter region that is bound by PnpR (Figure 5A) and PnpM (Figure 5B). After an approximately 270 bp 6-Fluorescein amidite (FAM)-labeled DNA fragment corresponding to the promoter region of *pnpC* was incubated with purified PnpM or PnpR, these complexes were treated with DNase I, followed by DNA fragment analysis by capillary electrophoresis. In the absence of PNP, PnpM protected a continuous 27 bp region that had also been determined to be from −88 to −62 relative to the *pnpCDEFG* TSS. The protection sequences contained an imperfect inverted repeat GTT-N_11_-AAC motif, which was similar to the T-N_11_-A motif of LTTRs consensus binding sequences, RBS, with positions −80 to −64 relative to the TSS of the *pnpCDEFG* operon. After analyzing the region of *pnpC* promoter, it was found that PnpM was bound to the sequence of putative RBS GTT-CGCGTTTCGCA-AAC in the promoter region of *pnpCDEFG* (Figure 5C). In the region of promoter *pnpA1M*, a similar putative sequence GTT-CGCGAAAGGCA-AAC was also present (Figure 5D). The protection region of PnpM was significantly increased from above a 27 bp sequence to 38 bp sequence by PnpM in the presence of PNP, and extended to the −51 region. This data tentatively suggested that the protection of extended 11 bp, from −61 to −51 relative to the TSS, and was the putative ABS (Figures 5B,C). The regulator PnpR shared the same 27 bp region DNA-binding regions of promoter *pnpCDEFG* with PnpM in the absence of the inducer (Figure 5A). The protection region was increased from 27 to 39 bp sequence by PnpR in the presence of PNP (Figure 5C).

### Effect of the different substrates on the promoter activity of *pnpC* promoter

**Figure 6:**
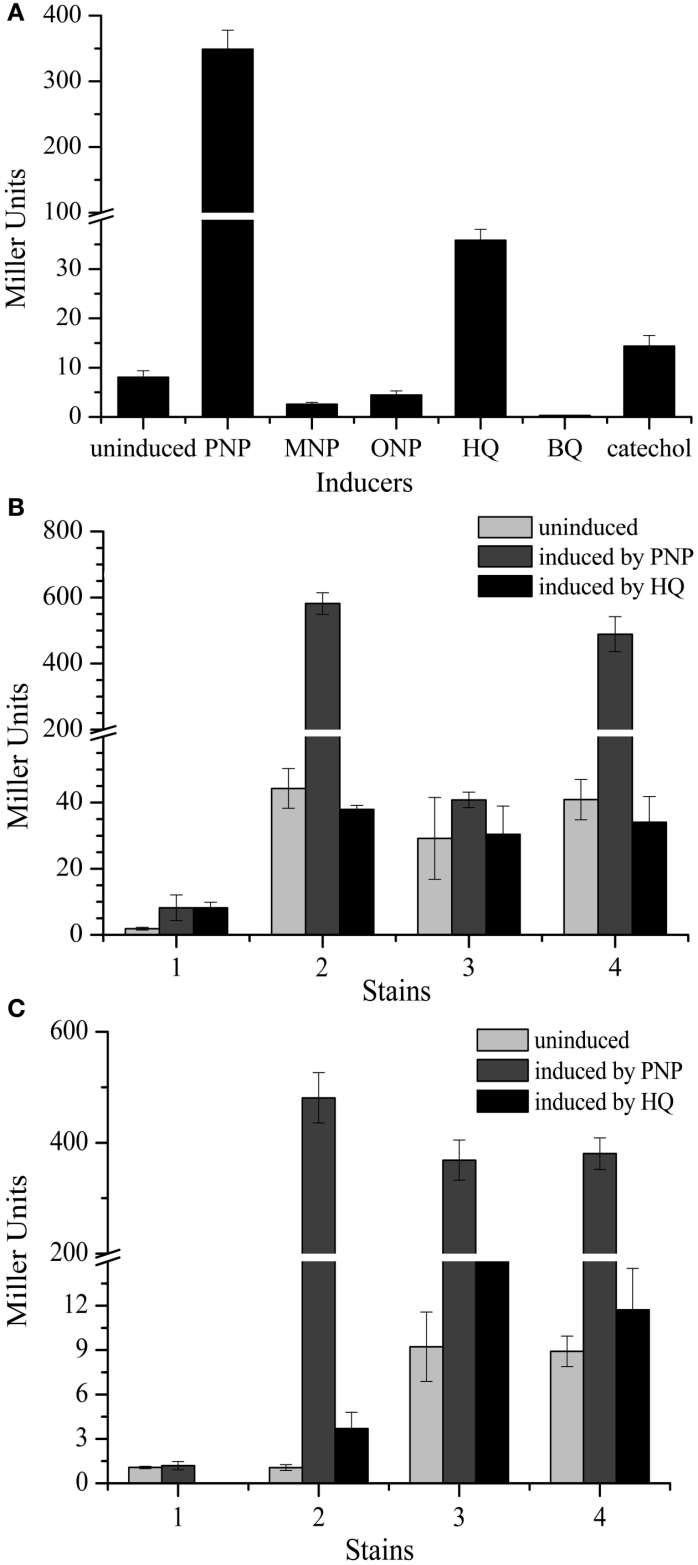
Determination of promoter activities by β-galactosidase. **(A)** Determination of promoter activities of *pnpC* operons induced by different substance. The β-galactosidase activity was determined in the strain PaW340 containing constructs pCM*gfp*-lpC*lacZ* and pBBR1-*tacexpnpM*. The cells were induced by the following substrates: *p*-nitrophenol (PNP), *m*-nitrophenol (MNP), *o*-nitrophenol (ONP), hydroquinone (HQ), benzoquinone (BQ) and catechol, respectively. There was a significant difference in promoter activity between PNP and HQ as well as other substrates as inducers (*p* < 0.05). **(B)** Expression of promoter *pnpA*-*lacZ* translational fusions in strain PaW340. The β-galactosidase activities were determined in the strains: 1. PaW340 (pCM*gfp*-lpA*lacZ*+pBBR1-mcs2); 2. PaW340 (pCM*gfp*-lpA*lacZ*+pBBR1-tac*pnpR*); 3. PaW340 (pCM*gfp*-lpA*lacZ*+pBBR1-tacex*pnpM*); 4. PaW340 (pCM*gfp*-lpA*lacZ*+pBBR1-tac*pnpMpnpR*). There was a significant difference in the promoter activity of *pnpA* operon between regulator PnpR and PnpM with or without PNP (*p* < 0.05, Paired-samples test). **(C)** Expression of promoter *pnpC*-*lacZ* translational fusions in strain PaW340. The β-galactosidase activities were determined in the above strains: 1. PaW340 (pCM*gfp*-lpC*lacZ*+pBBR1-mcs2); 2. PaW340 (pCM*gfp*-lpC*lacZ*+pBBR1-tac*pnpR*); 3. PaW340 (pCM*gfp*-lpC*lacZ*+pBBR1-tacex*pnpM*); 4. PaW340 (pCM*gfp*-lpC*lacZ*+pBBR1-tac*pnpMpnpR*). There was a significant difference in the promoter activity of *pnpC* operon among different inducer (*p* < 0.05, Paired-samples test). Light gray columns stand for gene expression level without inducer. Gray columns stand for gene expression level induced by PNP. Black columns stand for gene expression level induced by HQ.

To further investigate *pnpC* promoter activity, the constructs pCM*gfp*-lpC*lacZ* and pBBR1-*tacexpnpM* were transformed into strain PaW340 to measure *pnpC* promoter activities in response to various substrates. The expression of β-galactosidase activity in strain PaW340 was induced by following substrates: PNP, *m*-nitrophenol (MNP), *o*-nitrophenol (ONP), HQ, BQ and catechol, the final concentration of these six substrates was 0.3 mM for each one. No activity was detected except when PNP and HQ was used as inducers. The β-galactosidase activity of PNP-induced cells was 348.7- and HQ-induced cells was 35.8-fold higher (*P* < 0.05) than in non-induced cells, respectively (Figure 6A). These results were in agreement with *in vivo pnpC* expression measurements, further demonstrating that the major inducer for the promoter activity was PNP.

### Determination of promoter activity of *pnpC* and *pnpA* promoters

To further investigate *pnpC* promoter activity, pCM*gfp*-lpC*lacZ* was used to see if *cis*-acting DNA sequences was required for the correct regulation of the expression of *pnpR* or *pnpM* or both in strain PaW340 carrying pBBR1-*tacpnpR* or pBBR1-*tacexpnpM* or pBBR1-*tacpnpMpnpR*. In addition, the above constructs with a promoter region were introduced in strain PaW340 carrying pBBR1mcs-2 as the negative controls. In the absence of PNP, the LacZ expression levels in the constructs with 270-bp corresponding *pnpC* promoter region were all evidently low. In the presence of PNP, the expression levels of LacZ were higher by 406-, 306-, or 316-fold in strain PaW340 carrying pCM*gfp*-lpC*lacZ* plus pBBR1-*tacpnpR* or pBBR1-*tacexpnpM* or pBBR1-*tacpnpMpnpR*. In the presence of HQ, the expression levels of *Lac*Z were higher by 3.6-, 88-, and 11-fold (Figure 6C), respectively. In order to test *pnpA* promoter activity with different regulators, strain PaW340 carrying pCM*gfp*-lpA*lacZ* (containing approximately 300-bp *pnpA* promoter-*lacZ* translational fusion) with above three individual constructs (pBBR1-*tacpnpR* or pBBR1-*tacexpnpM* or pBBR1-*tacpnpMpnpR*) was used. It can be concluded from Figure 6B that PNP-induced *Lac*Z activity driven by the *pnpA* promoter were significantly increased only in the presence of *pnpR* but not *pnpM*. Therefore, these showed that only PnpR was involved in the transcriptional activation of *pnpA*, while PnpR and PnpM were both involved in the transcriptional activation of P*pnpC*.

## Discussion

In our previous work, an LTTR PnpR was found to activate the transcription of four operons of *pnpA, pnpB, pnpCDEFG*, and *pnpR* involved in the PNP degradation by *Pseudomonas* sp. strain WBC-3 (Zhang et al., 2015). Mutation of the four promoters abolished the binding capability of purified PnpR. Moreover, the promoter activities were completely lost except for P*pnpC*. These observations led us to investigate the possible involvement of an additional regulator in the transcriptional activation of the *pnpCDEFG* operon. In the present work, an LTTR PnpM with 44% identity to PnpR was identified as an activator for the *pnpCDEFG* operon encoding HQ mineralization in the PNP catabolism, in addition to PnpR. This hypothesis was based on our previous study and has been addressed in the current study. Thus, we conclude that the transcriptional regulation of PNP catabolism in strain WBC-3 is triggered by both LTTRs PnpR and PnpM. PnpR activates *pnpA* and *pnpB* operons encoding the initial monooxygenation and reduction, and both PnpR and PnpM are involved in the transcriptional activation of the *pnpCDEFG* operon encoding the ring-cleavage of HQ and beyond. The principal inducer for all three catabolic operons is PNP and HQ might be a much weaker inducer and for the *pnpCDEFG* operon only.

With regard to other PNP utilizers, it was previously reported that an LTTR PnpR_DLL−E4_, with 85% identity to PnpM was found to be involved in the regulation of HQ degradation in *Pseudomonas putida* DLL-E4 (Shen et al., 2010; Chen et al., 2016). Unlike *pnpM, pnpR*_DLL−E4_ is adjacent to the HQ catabolic cluster *pnpC1C2DECX1X2*. However, no *in vitro* analysis of PnpR_DLL−E_ was performed, such as EMSA, footprinting or promoter activity analysis. If the regulation system in strain DLL-E4 is similar to the one in strain WBC-3, genes homologous to *pnpR* from strain WBC-3 should be present in strain DLL-E4 but it has not been found so far. HQ is an important metabolite during aromatic catabolism and its pathway also exists as a downstream pathway in other aromatic degradations than PNP, such as alkylphenols degradation in strain *Sphingomonas* sp. strain TTNP3 (Kolvenbach et al., 2012); 4-hydroxyacetophenone degradation in *Pseudomonas fluorescens* ACB (Moonen et al., 2008); and 4-Fluorophenol degradation in *Arthrobacter* sp. strain IF1 (Ferreira et al., 2009). Of these bacterial strains, gene clusters similar to *pnpCDEFG* encoding HQ degradation are indeed present and putative regulators were also found to be nearby the HQ degradation gene clusters in some cases. For example, a putative AraC-type transcriptional regulator encoding-gene *hqdR* was next to the gene encoding hydroquinone-1,2-dioxygenase involved in the degradation of alkylphenols in *Sphingomonas* sp. strain TTNP3 (Kolvenbach et al., 2012). Nevertheless, no regulation study for HQ degradation has been reported in any bacterial strain.

In this study, two LysR transcriptional regulators PnpM and PnpR (44% identity) were shown to be involved in the activation of PNP catabolism in the strain WBC-3 *in vitro*, with both playing overlapping roles in the expression of *pnpCDEFG* as proposed in Figure 1B. One of well-studied examples for the involvement of multiple regulators is that of two LTTRs BenM and CatM which are complex regulatory circuits involved in benzoate consumption by *Acinetobacter baylyi* ADP1. Although both BenM and CatM activated the transcription of *benABCDE* operon, they responded to different inducers (Bundy et al., 2002; Ezezika et al., 2006, 2007; Craven et al., 2009). However, in this study, PnpR and PnpM both respond to the same inducer PNP (Figure 6C). In Gram-positive strain *Corynebacterium glutamicum*, two regulators GenR and GlxR are involved in the regulation of 3-hydroxybenzoate catabolism via gentisate. GenR is an IclR-Type specific regulator and GlxR is a CRP/FNR-type global regulator (Chao and Zhou, 2013, 2014). In contrast, PnpR and PnpM are both LysR-type specific regulators in this study. Although PnpM is a specific regulator for *pnpCDEFG* expression, it cannot singly function in the activation of all catabolic operons for PNP catabolism. Indeed, both PnpR and PnpM were involved in the intermediate HQ degradation and the starting compound PNP was the principal inducer rather than the substrate HQ for the enzymes *pnpCDEFG* encoded, since the latter was a much weaker inducer than the former in this system. This suggested that only HQ derived from PNP could then be further degraded by this system, and PNP degradation was sequentially activated by PnpR and PnpM in the presence of PNP. The observation of the incapability of strain WBC-3 growing on HQ is also clear and simple evidence for the above conclusion. However, it seems that the role of PnpR for *pnpCDEFG* operon can be replaced by PnpM *in vitro*, and it is unclear whether these two regulators may be in competition or act in a synergistic manner for *pnpCDEFG* promoter *in vivo*. This will require further efforts (such as chromatin immunoprecipitation assay) for a definite elucidation.

From the organization of *pnp* cluster in strain WBC-3, it can be tentatively concluded that the three operons may have evolved from different origins through patchwork assembly, each with their own promoters or regulators. Therefore, the complete degradation of PNP was regulated by two regulators, PnpR and PnpM, controlling three operons in total. Such complicated system for a single catabolic pathway may not be optimum and probably is an intermediate form during the adaptive evolution for PNP degradation. Indeed, most studied Gram-negative PNP utilizers were found to contain such three operons (Zhang et al., 2009, 2012; Shen et al., 2010; Wei et al., 2010), but with a recent, rare exception that the entire *pnp* genes are organized in a single operon in Gram-negative *Burkholderia* sp. strain SJ98, although they are very similar to their orthologous in strain WBC-3 (Min et al., 2014). Therefore, it can be reasonably considered that the transcriptional regulation of this single catabolic operon in strain SJ98 is less complex.

Usually, LTTRs bind to their regulated promoters at two sites regardless of the presence of relevant inducers: a strong RBS near position −65; and a weak ABS near position −35 (Schell, 1993; Porrua et al., 2007). In this study, the ABSs appeared from footprinting analyses (a 12-bp sequence binding with PnpR or a 11-bp sequence binding with PnpM only in the presence of the inducer PNP, as shown in Figure 5) may play a crucial role in activating the expression of *pnpCDEFG*. Although this observation is not common among LTTRs, several similar cases have been reported on the extended protection with relevant inducers. For instance, in the presence of an inducer, an additional 14 bp was protected by CatR, the regulator of catechol-degrading in *Pseudomonas putida* (Parsek et al., 1994, 1995). In *Pseudomonas aeruginosa*, the protection was extended 20 bp by Trpl for regulating tryptophan biosynthesis with an inducer (Chang and Crawford, 1990). The LigR-binding regions of the *ligK* promoter in *Sphingobium* sp. strain SYK-6 was extended by 16 bp (Kamimura et al., 2010). Interestingly, unlike the binding behavior of PnpR with the *pnpC* promoter, the binding region of promoters *pnpA* or *pnpB* by PnpR was unchanged with or without PNP (Zhang et al., 2015), indicating that the binding region was not only dependent on the regulator but also on its binding sequence. For most LysR proteins, the protein-DNA interaction region was shortened with the inducer. The mechanism of well-studied LTTRs, including AtzR, CbbR, OccR, OxyR, HadR, and QusR, was known as the sliding dimer model. These LTTRs cause the inducer-dependent shortening of the protected region from positions −80 to −20 to positions −80 to −30 and then a relaxation of the DNA bending. This was thought to be important in the release of the recognition site of RNA polymerase and the conformational change of regulator-DNA complex suitable for transcriptional activation (Toledano et al., 1994; Wang and Winans, 1995a,b; van Keulen et al., 2003; Porrua et al., 2007; Torii et al., 2013; Kubota et al., 2014). Nevertheless, in this study, the behavior of PnpM toward the *pnpCDEFG* promoter is somewhat different from the sliding dimer model for most LTTRs. The manner of DNA protection from PnpM binding to the *pnpCDEFG* promoter resembles the mentioned CatR, TrpI, or LigR, and the extended binding region in response to an inducer appears to be important for transcriptional activation.

## Author contributions

Conceived and designed the experiments: JW, WZ, and NZ. Performed the experiments: JW and WZ. Analyzed the data: JW, WZ, and HC. Wrote the paper: JW and NZ.

### Conflict of interest statement

The authors declare that the research was conducted in the absence of any commercial or financial relationships that could be construed as a potential conflict of interest.

## Abbreviations

PNP: *para*-nitrophenol
HQ: hydroquinone
BQ: *p*-benzoquinone.
