# Supplementary material for: PnpM, a LysR-Type Transcriptional Regulator Activates the Hydroquinone Pathway in para-Nitrophenol Degradation in Pseudomonas sp. Strain WBC-3
Source: Front Microbiol. 2017 Sep 14;8:1714. doi: 10.3389/fmicb.2017.01714 (PMC5603801; doi:10.3389/fmicb.2017.01714)
Supplement: Supplementary file 1 [file DataSheet1.docx]

Supplementary Material

**PnpM, a LysR-Type Transcriptional Regulator Activates the Hydroquinone Pathway in** ***para*-Nitrophenol Degradation in** ***Pseudomonas*** **sp. Strain WBC-3**

Jin-Pei Wang^1,3^, Wen-Mao Zhang^1^, Hong-Jun Chao^1^and Ning-Yi Zhou^1,2*^

^1^ Wuhan Institute of Virology, Chinese Academy of Sciences, Wuhan, China

^2^State Key Laboratory of Microbial Metabolism and School of Life Sciences & Biotechnology, Shanghai Jiao Tong University, Shanghai, China

^3^University of Chinese Academy of Sciences, Beijing, China

*** Correspondence:** Ning-Yi Zhou, Email: ningyi.zhou@sjtu.edu.cn

# Supplementary Figures


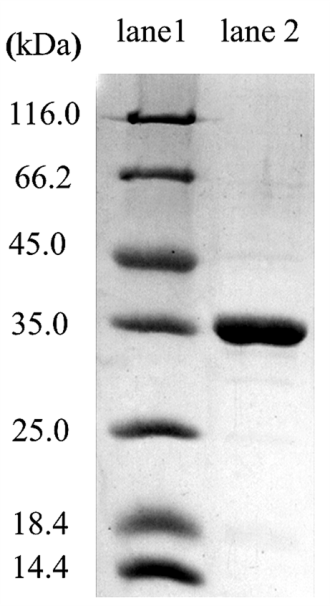


**Fig. S1. SDS-PAGE of purified PnpM-His_6_.**

Lane 1: Molecular mass standards (size in kDa are shown on the left); Lane 2: purified PnpM-His_6._





**Fig. S2.** **Gel filtration of PnpM with and without PNP**

A. The peak volume of protein blue dextran 2000 and PnpM is **64.22** ml in the absence of PNP.

B. The peak volume of protein blue dextran 2000 and PnpM is **63.84** ml in the presence of PNP.

C. The peak volume of native molecular mass of standard proteins carbonic anhydrase (29 KDa), bovine serum albumin (66 KDa), alcohol dehydrogenase from yeast (**150 KDa**), β-amylase from sweet potato (200 KDa), apoferritin from horse spleen (443 KDa), bovine thyroglobulin (669 KDa) are 87.09, 72.07, **64.53**, 59.96, 53.32, and 48.21ml, respectively.


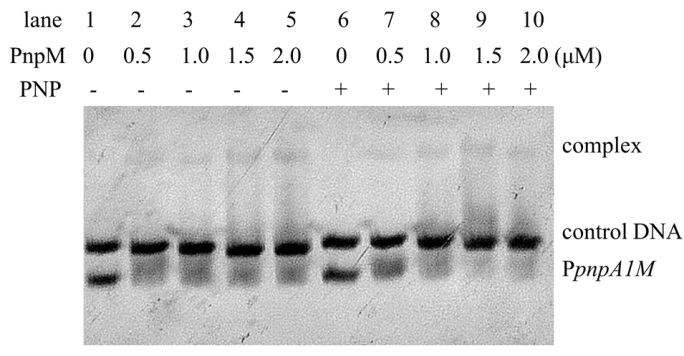


**Fig. S3. Electrophoretic mobility shift assays of PnpM binding with *pnpA1M* promoter (P*pnpA1M*).** PnpM binding with P*pnpA1M* with PNP or without PNP. +, stands for with PNP, ­−, stands for without PNP. Lanes 1-5 contain 0.03 μM DNA, with 0 μM, 0.5 μM, 1.0 μM, 1.5 μM, and 2.0 μM PnpM, respectively; lanes 6-10 contain 0.03 μM DNA and 0.3 mM PNP ,with 0 μM, 0.5 μM, 1.0 μM, 1.5 μM, and 2.0 μM PnpM, respectively. An approximately 400 bp DNA fragment *gfp*-1 was used as a control DNA (0.03 μM). Free probe is a fragment P*pnpA1M* of about 262 bp.

**Table S1 Primers in this study**

| Primer | Sequence (5′ to 3′) | Purpose |
| --- | --- | --- |
| For Disruption |  |  |
| EcoRI-pnpM-up-F | CGGAATTCGTCCGACGACAACGGCGTGAGC | To amplify upstream fragment  of *pnpM* of WBC-3 for gene knockout |
| pnpM-up-R | CAGAGATTTTGAGACAGGGGTGTTTCCGTCGTTGAGGGC |  |
| pnpM-down-F | GCTCGATGAGTTTTTCTAATGGCTGCCTGAAGCGGCCT | To amplify downstream fragment  of *pnpM* of WBC-3 for gene knockout |
| BamHI-pnpM-down-R | CGGGATCCGCGGTTATTGGCGAATCAAATGGAA |  |
| Infusion-kan-F | GCCCTCAACGACGGAAACACCCCTGTCTCAAAATCTCTGATGTTAC | \| To amplify kanamycin resistance \| \| --- \| \| gene for gene knockout \| |
| Infusion-kan -R | GAGGCCGCTTCAGGCAGCCATTAGAAAAACTCATCGAGCATC |  |
| PpnpA1M-F | CAACTTAAGGACCACGGCATATGCCGACG | To amplify *pnpM* with the promoter  of WBC-3 for gene complemetation |
| PpnpA1M-R | GATGCTGAGTAAACGATCCATGTATGCCCTCTGAATA |  |
| Infusion-pnpM-F | ATTCAGAGGGCATACATGGATCGTTTACTCAGCA |  |
| Infusion-pnpM-R | CAGGATCCTCAGCAAACCCAACCCCG |  |
| For EMSAs and *lacZ* fusion |  |  |
| gfp1-F | TTCAATGCTTTGCGAGATAC | To amplify 400 bp *gfp* as control DNA  for EMSAs |
| gfp1-R | AGGTAATGGTTGTCTGGTAAAA |  |
| lpA-F | ACATGCATGCGGCTTATGTGTGCGTGGCAC | To amplify 283bp *pnpA* promoter  for EMSAs and *lacZ* fusion |
| lpA-R | CGGGATCCCATTTTATTTTTCCTATAGAAAAC |  |
| lpB-F | ACATGCATGCTTCCTATAGAAAACGGCTCG | To amplify 334 bp *pnpB* promoter  for EMSAs and *lacZ* fusion |
| lpB-R | CGGGATCCCATGTCGGTTTCCTTGATAA |  |
| lpC-F | ACATGCATGCATTCGGACTGGTCGATAAAC | To amplify 270 bp *pnpC* promoter  for EMSAs and *lacZ* fusion |
| lpC-R | CGGGATCCCATGATTTCACCTTTTTTGTTGTAA |  |
| pA1M-F | ACATGCATGCCCCGACGCCTCTGACTTTTATTG | To amplify 262 bp *pnpA1* promoter  for EMSAs and *lacZ* fusion |
| pA1M-R | CGGGATCCCATGTATGCCCTCTGAATAACGC |  |
|  |  |  |
| pCm-F | ACATGCATGCCTGTGCTGTTGATC**AAA**CGCGTTTCGCAAACAC | To amplify mutation *pnpC* promoter  for *lacZ* fusion |
| For expression |  |  |
| pET30a-pnpM-F | GGAACACATATGGATCGTTTACTCAGC | To amplify *pnpM* gene of WBC-3  for expression in *E.coli* |
| pET30a-pnpM-R | TCAAGCTTGCAAACCCAACCCC |  |
| ptac-F | AAGGTACCGACTGCACGGTGCACCAATG | To amplify *tac* promoter  for expression in strain PaW340 |
| ptac-R | CGGGATCCCTTAAAGTTAAACAAAATTATTTCTAG |  |
| ppnpA1-F | CGGGATCCCCCGACGCCTCTGACTTTTATTG | To amplify *pnpM* with the promoter  for expression in strain PaW340 |
| ppnpA1-R | CTCGCGAAGGTCGTCAACGAGTATGCCCTCTGAATAACGC |  |
| expnpM-F | GCATTTCATCGTCACTGCTCGTTGACGACCTTCGC |  |
| expnpM-R | CCGAGCTCTTAATGATGATGATGATGGTGGCAAACCCAACCCCGCTC |  |
| tac-pnpM-F | AGGAATTCGACTGCACGGTGCACCA | To amplify *pnpM and pnpR* with the *tac* promoter for expression in strain PaW340 |
| tac-pnpM-R | ACGGATCCTCAGCAAACCCAACCC |  |
| tac-pnpR-F | CGGGTACCGAAAGGTTTTGCACCATTCG |  |
| tac-pnpR-R | CCGAGCTCTTATTGCTCTTCCTGTTCCGG |  |
| For RT-qPCR |  |  |
| qRT-16s-F | TTGACGTTACCGACAGAATAAGC | \| To amplify a 157 bp fragment of \| \| --- \| \| 16S rDNA of WBC-3 for RT-qPCR \| |
| qRT-16s-R | TGCAGTTCCCAGGTTGAGC |  |
| qRT-pnpA-F | GCACTGAAACTGGGTAAAGC | \| To amplify a 181 bp fragment of \| \| --- \| \| *pnpA* of WBC-3 for RT-qPCR \| |
| qRT-pnpA-R | CATGGCGATAGGCGAAATCC |  |
| qRT-pnpB-F | CGGAACTGATGCCTGAAGAG | \| To amplify a 142 bp fragment of \| \| --- \| \| *pnpB* of WBC-3 for RT-qPC \| |
| qRT-pnpB-R | GGCGTTGCGTAAGGGATGCT |  |
| qRT-pnpC-F | CTTCGCCTCGCTGGATAAC | \| To amplify a 145 bp fragment of \| \| --- \| \| *pnpC* of WBC-3 for RT-qPCR \| |
| qRT-pnpC-R | GTAGCCGAGGTTCAGGCC |  |
| qRT-pnpR-F | AAGCGGGTAACTTCTCCAAC | \| To amplify a 128 bp fragment of \| \| --- \| \| *pnpR* of WBC-3 for RT-qPCR \| |
| qRT-pnpR-R | GAGATGCCCACTTCAGACAG |  |
| qRT-pnpM-F | TGCAGGAAGGCTACGACATC | \| To amplify a 139 bp fragment of \| \| --- \| \| *pnpM* of WBC-3 for RT-qPCR \| |
| qRT-pnpM-R | GGCGGGTGATTGCTAAGG |  |
| For 5′-RACE |  |  |
| pnpA1M-GSP2 | GCCCGTGCCTCAAGTACC | \|  \| \| --- \| \| To amplify cDNA of WBC-3 for 5′-RACE \| |
| pnpA1M-GSP3 | CCCTGCCCAGTTTCAATG |  |
| For RT-PCR |  |  |
| RT-pnpR-F | TCTGCTTGGCGGCGTTGGAG | \| To amplify 501 bp of the *pnpR-pnpA1* \| \| --- \| \| spanning region of WBC-3 \| |
| RT-pnpA1-R | GATACCCGCCCTGCCCAGTTTC |  |
| RT-pnpA1-F | ATCGGGGAGGTGGTGAAGCA | \| To amplify 419 bp of the *pnpA1-pnpM* \| \| --- \| \| spanning region of WBC-3 \| |
| RT-pnpM-R | ATGGAACAGCGGCGCATTG |  |

The restriction site is underlined. Oligonucleotide positions altered from the template sequences are in boldface.
